# Comparison of arthroscopic internal drainage and open excision for the treatment of popliteal cysts

**DOI:** 10.1186/s12891-022-05658-2

**Published:** 2022-07-30

**Authors:** Chao You, Zhen Cheng, Yongjie Xia, Chao Deng, Yibiao Zhou

**Affiliations:** 1 Department of Orthopedics, Shenzhen Children’s Hospital, Shenzhen, Guangdong Province China; 2 China Medical University, Shenyang, Liaoning Province China

**Keywords:** Arthroscopy, Popliteal cyst, Children, Minimally invasive, Internal drainage

## Abstract

**Background:**

The purpose of this study was to introduce the arthroscopic internal drainage with anterior-anteromedial approach for the treatment of popliteal cysts in children. To compare its clinical efficacy with open surgery.

**Methods:**

This was a retrospective case–control study of 102 patients diagnosed with popliteal cysts from January 2018 to February 2020 who received surgery. The study included 27 cases with minimally invasive group (MI group) and 75 cases with open surgery group (OS group). The MI group included 21 males and 6 females, age 6.71 ± 2.16 years who received arthroscopic internal drainage of the cysts to adequately widen the valve opening between the cyst and the articular cavity, excised the fibrous diaphragm without complete excision of the cyst wall. The OS group included 57 males and 18 females, age 6.21 ± 1.67 years who received open excision. The clinical parameters regarding the preoperative characteristics and surgical results were compared. Ultrasound or MRI was used to identify the recurrence of the popliteal cysts. Rauschning-Lindgren grade was recorded to evaluate the clinical outcome.

**Results:**

All patients were followed up for at least 24 months. There were no significant differences between the two groups in age, gender, left and right sides, disease time, cyst size, length of hospitalization, preoperative Rauschning-Lindgren grade (*p* > 0.05). At the last follow-up, the preoperative and postoperative Rauschning-Lindgren grade was improved in both groups. Compared with the OS group, operation time was significantly shortened in the MI group (28.89 ± 4.51 min vs 52.96 ± 29.72 min, *p* < 0.05). The MI group was superior to the OS group in terms of blood loss and plaster fixation, with statistical significance (*p* < 0.05). There was obvious difference in recurrence rate between the two groups (0% vs 17.33%, *p* = 0.018). No postoperative complications occurred during the follow-up period.

**Conclusions:**

Compared with open excision, the treatment of popliteal cyst in children by arthroscopic internal drainage to expand the articular cavity and eliminate the “one-way valve” mechanism between the cyst and the articular cavity exhibits better clinical outcomes and significantly reduces the recurrence rate, which is worthy of further clinical promotion.

## Background

Popliteal cyst, the general term for popliteal synovial cyst, is a common knee joint disease, which was first described as enlargement of synovial fluid in the popliteal fossa by Baker in 1877, mostly occurring between the semimembranous tendon and the medial head of gastrocnemius muscle [1]. Clinically, they typically present with palpable masses in the popliteal fossa, with or without knee pain and limited movement. Popliteal cysts may be divided into primary and secondary cysts based on the onset of age and the different pathogenesis. Primary popliteal cyst, the lesion is typically unilateral, and is more common for boys and mostly occurs in children under 15 years [2]. For children, Baker’s cyst is seldom associated with intra-articular pathology and generally has no obvious symptoms. Baker’s cyst is most commonly an incidental finding during physical examination. Occasional discomfort may occur when cyst enlargement becomes palpable. However, when cysts are combined with intra-articular lesions (meniscus tear, exudative osteochondritis, and so on), the symptoms of popliteal fossa swelling, pain, and limited joint flexion and extension activities are presented. Secondary popliteal cysts are most common in adults, often associated with intra-articular pathology, and communication exists between the bursa and the knee joint, articular fluid can fill the cyst and a pathologic joint process can be transmitted to the bursa [3]. Typical manifestations are pain behind the knee joint, palpable local masses, and a feeling of distention in the popliteal fossa area. Sansone [4] reported that Baker’s cyst has been associated with one or more other pathology; the most common was a meniscal tear (83%), followed by chondral lesions, and anterior cruciate ligament tears. Johnson and colleagues [5] described the frequency of different kinds of intra-articular lesions in patients with popliteal cysts including osteoarthritis (81%), medial meniscus tear (68%), loose body (38%), edema (35%), and patellofemoral cartilage injury (30%).

Although the pathogenesis of Baker’s cyst is still unclear, it is generally believed that the valve mechanism plays a critical role in the formation. Some studies generally believed that the damage of intra-articular band and plica blocks the channels, leading to joint effusion entering into the synovial sac. However, it cannot flow into the articular cavity from the synovial sac, forming the “one-way valve” mechanism. The "one-way valve" mechanism is the reason for the formation, persistence and recurrence of cysts [6, 7]. Open-prone posterior cyst excision cannot effectively solve the problem of recurrence, which does not consider the intra-articular pathology and unidirectional valve mechanism. With the continuous progress of arthroscopic technology, arthroscopic treatment of popliteal cyst in adults has achieved satisfactory clinical efficacy [8–10]. But, posterior open resection of popliteal cyst is still used in children, and arthroscopic treatment is rarely reported until now.

The purpose of this study was to introduce an arthroscopic treatment technique for Baker’s cyst in children using an anterior-anteromedial approach. The authors hypothesized that patients who underwent arthroscopy would experience better clinical efficacy and lower recurrence rates compared to patients who underwent open excision.

## Materials and methods

The protocol for the study has been approved by the Ethics Committee of our Institutional and conforms to the provisions of the Declaration of Helsinki.

### Patient demographics

**Fig. 1:**
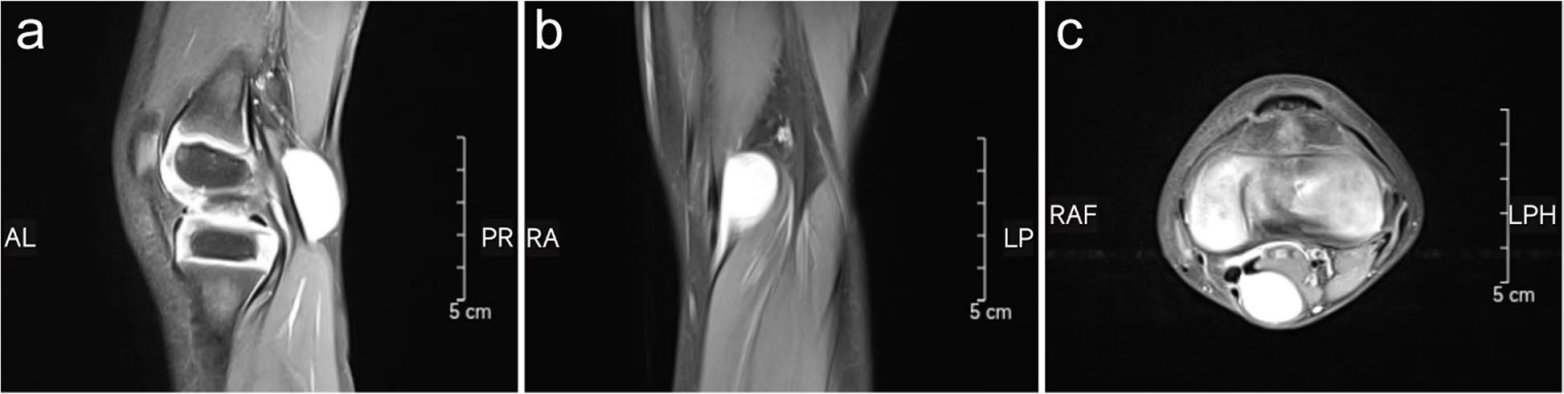
Pre-operative MR images of sagittal T2 (**a**), axial (**b**) and coronal view (**c**) demonstrate a popliteal cyst on the left knee

A total of 102 children with Baker’s cyst who received surgery in our hospital from January 2018 to February 2020 were included. There were 78 men and 24 women. All the patients underwent ultrasound or MRI examination before surgery to confirm the diagnosis, and evaluate the intra-articular pathology (Fig. 1). Rauschning-Lindgren grade [11] as the criteria was adopted to evaluate the preoperative status and postoperative outcome. Twenty-seven cases were treated by arthroscopic internal drainage (21 males and 6 females, age 6.71 ± 2.16 years; grade 0 in 25 patients, grade I and grade II in one patient independently), all of which were unilateral, and 3 children had recurrence after open surgery among them. While 75 cases were treated by open excision (57 males and 18 females, age 6.21 ± 1.67 years; grade 0 in 66 patients, grade I in 6 patients and grade II in 3 patients), 70 patients had unilateral disease and 5 patients had bilateral. Respectively the basic clinical data of two groups of children, such as age, gender, left and right side, onset time, cyst diameter for statistical analysis, compare in operative time, the length of incision, intraoperative blood loss, plaster fixation and down time, hospitalization days. Recurrence and postoperative surgery-related complications were recorded during the follow-up visit.

### Statistical methods

SPSS (version 26.0, IBM Corporation, Chicago, USA) software was used for all statistical analyses. The data were presented as the mean ± standard deviation or median and inter-quartile range (IQR). Categorical variables were presented with absolute frequencies (n) and percentages (%). Independent t-test or Mann–Whitney U test was used to compare the statistical significance of differences in continuous data. The dichotomous variables were compared using the Pearson Chi-squared test or Fisher exact probability test. All statistical data were two-sided and were evaluated at the significance level of 5%, a *P*-value < 0.05 was considered statistically significant.

### Surgical technique

**Fig. 2:**
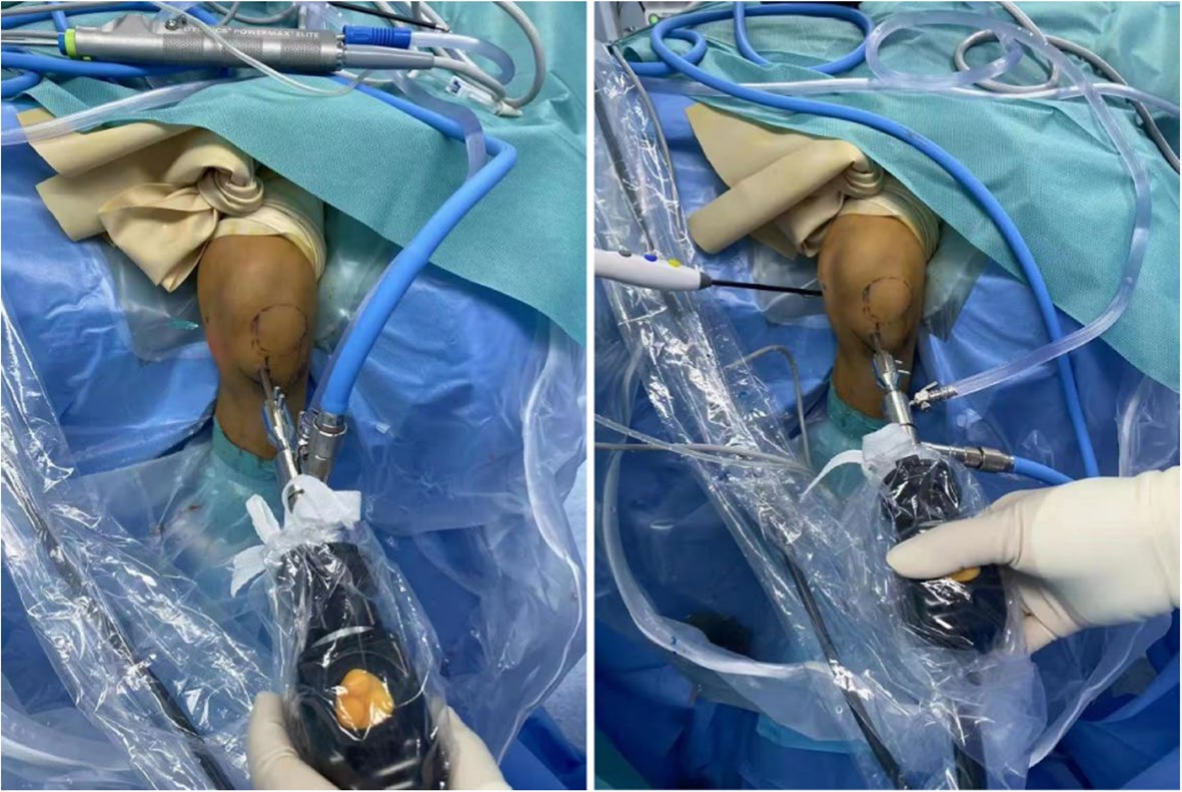
Two cooperated approach: anterior approach and medial approach

After satisfactory general anesthesia, the patient was placed in supine position with knee flexion at 90°. Routine disinfection and operation sheet, 1 ml methylene blue was injected into the cyst for intraoperative determination of valvular opening, and 10-20 ml normal saline was injected into the articular cavity. Tourniquet was applied to control bleeding during the procedure. An anterior-anteromedial approach has been established to detect and treat intra-articular lesions (such as meniscus tear, synovitis, and loose body). After that, the posteromedial compartment then entered with the lens through the space between the posterior cruciate ligament and the medial condyle of the femur (Fig. 2).

**Fig. 3:**
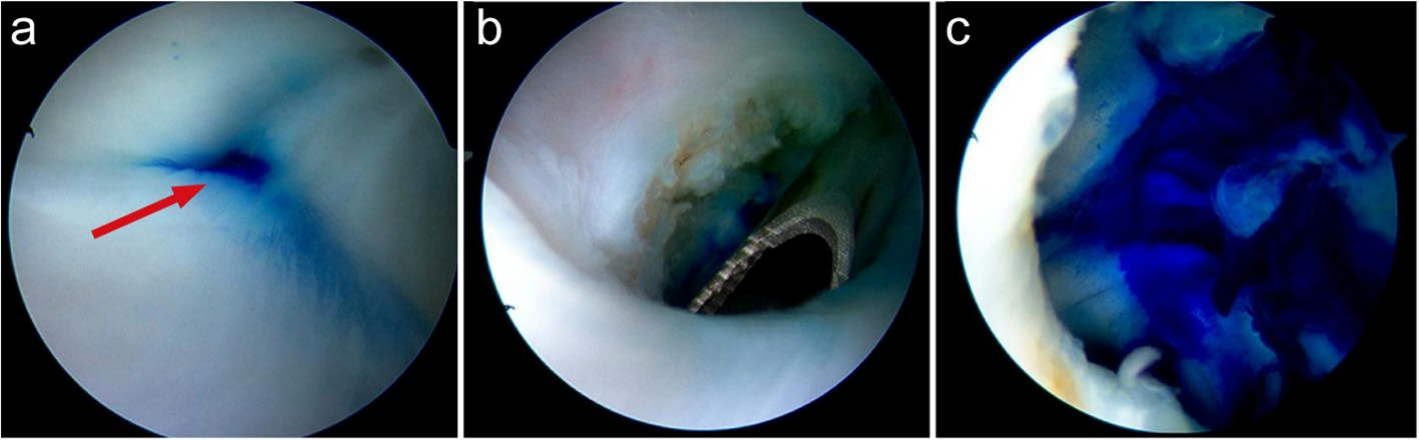
**a** Arthroscopic view of capsular in the posteromedial wall, the red arrow to the “one-way valve”; **b** Resection of the capsular folds to reestablish the two-way communication between articular cavity and the cyst; **c** The entire picture and internal condition of the cyst observed under arthroscopy

The weak area or fissure of Baker’s cyst in the posteromedial compartment joint capsule was explored to determine the position of the medial gastrocnemius tendon, the lateral folds of the posterior medial synovial membrane, and the cyst opening. A shaver and probe were used to find the opening of the cyst, while the opening was usually located at the posteromedial side of the medial head of the gastrocnemius which was behind the capsular fold (Fig. 3a). We then used a shaver to resect the capsular folds to open the cyst’s communication, and methylene blue was seen flowing into the joint from the cyst (Fig. 3b). Enter into the cyst deeply, capsule folds and internal septum were dissected with a shaver. We completely enlarged the valvular opening by the shaver to reestablish a normal bidirectional communication (Fig. 3c). After surgery, the joint cavity was irrigated and drained as much as possible, and the incision sutured and bandaging were carried out. Note that all the above operations were performed inside the medial head of the gastrocnemius muscle in order to avoid nerve and blood vessel damage.

After surgery, no plaster fixation was required, and walking with weight was allowed.

### Follow-up

After discharge, all patients underwent ultrasound or MRI examinations regularly in the outpatient department to check whether the Baker’s cyst had recurrence. Assessment of clinical outcome by Rauschning-Lindgren grading at the last follow-up.

## Results

A total of 102 children with Baker’s cyst were followed up for 2 years at least. Basic characteristics for patients in the two groups were listed in Table 1. There were no significant differences between the MI group and OS group in preoperative basic clinical data including age (*P* = 0.216), gender distribution (*P* = 0.852), cyst size (*P* = 0.051), onset time (*P* = 0.912) and R–L grade (*P* = 0.526).

**Table 1:** Basic characteristics in the MI group and the OS group

	MI group(*n* = 27)	OS group(*n* = 75)	*P* value
Age (years)	6.71 ± 2.16	6.21 ± 1.67	0.216
Gender(n, %)			0.852
male	21(77.8%)	57(76.0%)	
female	6(22.2%)	18(24.0%)	
Left and right sides(n, %)			0.478
left	17(63.0%)	42(56.0%)	
right	10(37.0%)	28(37.3%)	
double	0	5(6.7%)	
Onset time(months)	2.00(0.75, 12.00)	2.00(1.00, 6.00)	0.912
Cyst size(cm)	4.66 ± 1.95	4.43 ± 1.27	0.051
Pre-operation Rauschning-Lindgren grade			0.526
0	25(92.6%)	66(88.0%)	
I	1(3.7%)	6(8.0%)	
II	1(3.7%)	3(4.0%)	

As expected, in the aspect of operative time, the MI group (28.89 ± 4.51 min) is significantly shorter compared to the OS group (52.96 ± 29.72 min). In addition, the MI group was superior to the OS group in terms of length of incision (0.84 ± 0.08 cm), blood loss (Table 2; *P* < 0.05). Arthroscopic internal drainage technique was used for treatment, without plaster fixation after surgery, and early activities was possible. Nevertheless, there was no significant difference between the two groups in terms of total hospitalization time (4.70 ± 0.78 days vs 4.53 ± 0.93 days)(Table 2; *P* = 0.709). In the OS group, 13 patients had recurrence (17.3%) after surgery, which was markedly higher than that in the MI group (17.3% vs 0%, *P* = 0.018) (Table 2).

**Table 2:** The intraoperative situation and postoperative follow-up of the two groups

	MI group(*n* = 27)	OS group(*n* = 75)	*P* value
Surgical time (min)	28.89 ± 4.51	52.96 ± 29.72	< 0.000
Length of incision (cm)	0.84 ± 0.08	5.71 ± 1.21	< 0.000
Blood loss(ml)	1.00(1.00, 1.00)	10.00(9.00, 10.00)	< 0.000
Hospitalization time(d)	4.70 ± 0.78	4.53 ± 0.93	0.709
Recurrence			0.018
NO	27(100%)	62(82.7%)	
YES	0	13(17.3%)	

**Fig. 4:**
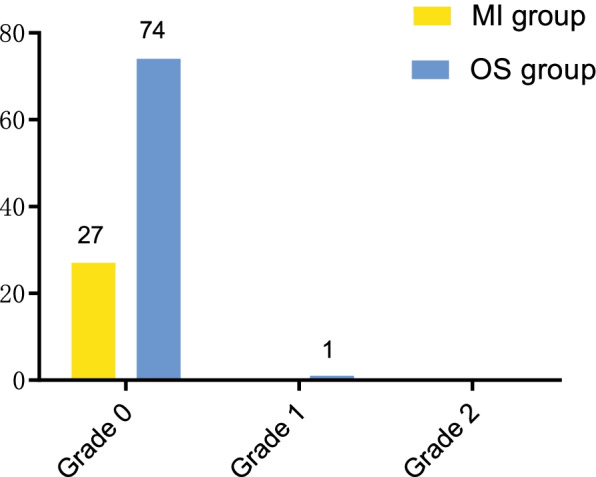
Distributions of Rauschning and Lindgren grade in the two groups at the last follow-up. Grade 0, no swelling and pain, no limitation of movement; Grade 1, slight swelling and pain following intense activity, minimal limitation of movement < 10°; Grade2, moderate swelling and pain following normal activity, 10–20° range limitation; Grade 3, considerable swelling and pain even at rest, > 20° range limitation

No postoperative complications were observed in all the children during follow-up. According to the Rauschning-Lindgren grade, the distributions of the MI group at the final follow-up were as follows: the number of grade 0 increased from 25 patients before surgery to 27 patients, grade I and grade II decreased to 0. The relevant findings in the OS group at the final follow-up were: grade 0 increased from 66 patients to 74, two patients for grade I and 0 for grade II (Fig. 4). There was no marked difference between the two groups (*P* > 0.05).

## Discussion

The necessity of surgical treament for popliteal cyst is controversial. Influenced by the concept of Medical environment in China, most parents prefer surgery for popliteal cyst, even though most children have no uncomfortable symptoms. This is why most of the children in our study who had a Rausching-Lindgren preoperative score of 0 were still operated on. Some scholars believe that Baker’s cyst in children does not need surgical treatment, and most of them can be spontaneously resolved or relieved [12–15]. However, some researchers have pointed out that surgical intervention is necessary when children have uncomfortable symptoms [16].

In this study, 102 patients were treated in our center from January 2018 to February 2020. The follow-up results showed that the joint function of the two groups recovered well after operation, and the Rauschning-Lindgren grade was improved obviously. In the OS group, 13 children recurred during the follow-up period with a recurrence rate of 17.3%, none of in the MI group(*p* = 0.018). It is reported that the recurrence rate after simple popliteal cystectomy in posterior open surgery is more than 40–50%. Because the intra-articular lesions are not solved, the synovial fluid will continue to exudate outward. Even if the stump is closed, with the increase of pressure, the stump rupture will eventually lead to recurrence. This situation is also seen in children [17]. We considered that open excision just relieve the “one-way valve”. This communication between the bursa and the joint cannot be completely closed by suturing, and the remnant of the cyst wall causes continuous production of cyst fluid, or the children do not cooperate after the operation, the suture falls off due to early activity, the “one-way valve” mechanism is formed again. The continuous one-way flow of fluid from the knee joint cavity to the cyst is an indispensable cause of recurrence [9]. In addition, open excision cannot solve the problem of intra-articular lesions at the same time and due to a wide range of surgical exposure, there is a risk of vascular or nerve injury [15, 18].

With the development of minimally invasive pediatric surgery, we realized that arthroscopic surgery can provide significant benefits for patient. In addition, open resection can not effectively solve the “one-way valve” mechanism, resulting in a high postoperative recurrence rate, which makes it necessary to propose a new treatment technology in children. If this mechanism is not eliminated, the recurrence rate will be high [6].

Thus, we conducted this study to introduct the arthroscopic treatment and analyze the advantages over open. Our center puts forward the technique of the arthroscopic internal drainage with anterior-anteromedial approach to expand the communication for the treatment. We encountered 27 cases were treated under arthroscopy at our hospital. Our findings showed the superiority of arthroscopic internal drainage to open excision in operative blood loss, the smallest incision, the operation time(*p* < 0.05). Arthroscopic surgery can provide significant benefits to patients, including smallest incision, less trauma and smaller scar after operation, which do not need plaster fixation, do not affect the function of knee joint, and reduce the pain caused by plaster on children. In fact, most scholars have reported that the treatment of adult popliteal cysts by arthroscopic enlargement or resection of the cyst wall and internal drainage instead of open excision has achieved satisfactory clinical results, and the recurrence rate has been greatly reduced. At the same time, because of popliteal cysts with intra-articular lesions, arthroscopic treatment can be used to explore and treat at the same time [9, 10, 18].

Whether the popliteal cysts are connected with the articular cavity is controversial. Studies by Malloch [19] have shown that cysts are not connected to the articular cavity. Gristina found that 48% of popliteal cysts in children under the age of 12 were connected to the joints, and they believed that pressure from the articular cavity might stimulate normal mucous cysts, causing them to expand and manifest as popliteal lumps [12]. Bohensky [20] argued that it is not necessary to close the channel between the cyst and the joint cavity. Because the research data show that there was good communication between the knee joint cavity and the cyst in about 50% of normal people, but no popliteal cyst was found [21]. And of the 1001 cases examined by MRI for various reasons, 4.7% to 37% were popliteal cysts, and in these cases, the cysts communicated with the articular cavity [4]. The author believes that the open excision is not necessary to close the communication between the bursa and the joint space, which cannot solve the problem of recurrence. Kongmalai et al. [22] have pointed out that the fibrous membrane, nodule and septa in the cyst will increase the recurrence rate of the popliteal cyst after arthroscopic cystectomy. In fact, in our research, most of the children have intracapsular septum, and shaver is usually used to clear so that the cyst can communicate fully with the knee joint during surgery. The mode of operation in this center is to expand the channel and eliminate the valve mechanism, and the joint function of children will not be affected after operation, which can effectively reduce the recurrence rate. Sansone and De Ponti [23] also showed that the enlargement of the cyst did not affect the joint structure. Therefore, arthroscopic internal drainage with anterior-anteromedial approach may be an effective technique for the treatment of popliteal cyst.

### Limitations of the study

Although our results show that the operation time of arthroscopy is better than that of open surgery, and there are no postoperative complications, but this is a retrospective case–control study and the early learning curve of arthroscopy is longer. Furthermore, the popliteal neurovascular bundle is usually located on the outside of the cyst, which is the main dangerous structure during the operation. When operating with a shaver, we should pay attention to avoiding damaging blood vessels and nerves, which requires high surgical techniques and the clinical experience of surgeons. Using PASS 20.0, the estimated sample size was 10 and 30 participants for MI and OS group respectively, based on confidence level (95%), power (90%), expected surgical time of popliteal cysts among MI group and OS group, with a ratio of controls to cases 3:1. Even if we increase the sample size to compensate for reliability, considering the current controversy about surgical treatment of Baker’s cyst in asymptomatic children, including arthroscopic treatment, we need longer follow-up time and larger sample size to determine the satisfactory clinical effect of arthroscopic surgery.

## Conclusions

Our results suggest that arthroscopic internal drainage with anterior-anteromedial approach to expand communication is effective and safe for treating Baker’s cyst, which shows satisfactory clinical results and lower recurrence rate. It not only clears the intra-articular lesions but also reestablishes the “two-way valve” mechanism between the bursa and the joint space. Compared with open excision, arthroscopic internal drainage has small incision, less bleeding, shorter operation time, no plaster fixation after operation, and can participate in social activities in the early stage. In addition, we propose a new clinical classification in the hope of providing evidence for subsequent clinical treatment. Although it may take longer time to master this technique, especially in children, we still recommend arthroscopic treatment for Baker’s cyst in clinical practice.

## Data Availability

The datasets used and analyzed during the current study are available from the corresponding author on reasonable request.
